# High-resolution *in situ* characterization of laser powder bed fusion via transmission X-ray microscopy at X-ray free-electron lasers

**DOI:** 10.1107/S1600577525001675

**Published:** 2025-04-01

**Authors:** Zane Taylor, Tharun Reddy, Lichao Fang, Patrick Oppermann, Patrick L. Kramer, Franz-Josef Decker, Matthew Seaberg, Matthieu Chollet, Tim van Driel, Alex Halavanau, Philip Hart, Matthew Dayton, Frank Seiboth, Wenxin Wang, Carolyn Gee, Abigail Wilson, Rachel Margraf-O’Neal, Gourab Chatterjee, Ying Chen, Ilana J.P. Molesky, Yifan Wang, Sara Irvine, Jade Stanton, Cynthia Melendrez, Kelsey Banta, Silke Nelson, Vivek Thampy, Kento Katagiri, Morten Haubro, Sen Liu, Dayeeta Pal, Lauren Moghimi, Christopher Tassone, Leora Dresselhaus-Marais

**Affiliations:** a Department of Materials Science and Engineering Stanford University Stanford CA94305 USA; b SLAC National Accelerator Laboratory Menlo Park CA94025 USA; c Linac Coherent Light Source SLAC National Accelerator Laboratory Menlo Park CA94025 USA; d Advanced hCMOS Systems, Albuquerque, NM87107, USA; e Center for X-ray and Nano Science (CXNS) Deutsches Elektronen-Synchrotron DESY Notkestr. 85 22607Hamburg Germany; f European X-ray Free-Electron Laser Facility, Holzkoppel 4, 22869Schenefeld, Germany; g Argonne National Laboratory Lemont IL60439 USA; h Department of Applied Physics Stanford University Stanford CA94305 USA; i Stanford Synchrotron Radiation Lightsource SLAC National Accelerator Laboratory Menlo Park CA94025 USA; j Department of Physics Technical University of Denmark Kongens Lyngby Denmark; k Department of Mechanical Engineering University of Louisiana at Lafayette Lafayette LA70503 USA; Australian Synchrotron, Australia

**Keywords:** laser powder bed fusion, LPBF, transmission X-ray microscopy, TXM, X-ray free-electron lasers, XFELs, *operando* techniques, high-resolution techniques

## Abstract

In this work, we describe the instrumentation used to perform the first *operando* transmission X-ray microscopy (TXM) and simultaneous X-ray diffraction of laser melting simulating laser powder bed fusion on the XCS instrument at the Linac Coherent Light Source (LCLS) X-ray free-electron laser (XFEL). Our TXM with 40× magnification in the X-ray regime at 11 keV gave spatial resolutions down to 940 nm per line pair, with effective pixel sizes down to 206 nm, image integration times of <100 fs, and frame rates tunable between 2.1 and 119 ns for two probe frames (0.48 GHz to 8.4 MHz). Images were recorded on Zyla and Icarus (UXI) detectors to trade off between spatial resolution and time dynamics. A 1 kW CW IR laser was coupled into the interaction point to conduct pump–probe studies of laser melting and solidification dynamics. Our temporal and spatial resolution with attenuation-based contrast exceeds that currently possible with synchrotron-based high-speed radiography. This system was sensitive to feature velocities of 10–12000 m s^−1^ but we did not observe any motion in this range in the laser melting of Al6061 alloy. Shockwaves were not observed and hot cracking proceeded at velocities below the detection limits. Pore accumulation was observed between successive shots, indicating that bubble escape mechanisms were not active. With proper experimental design, the spatial resolution, contrast and field of view could be further improved or modified. The increased brightness and narrower bandwidth of the XFEL allowed for this imaging technique and it lays the groundwork for a wide range of *operando* techniques to study additive manufacturing.

## Abstract 2

The first implementation of an *operando* transmission X-ray microscopy to study laser melting at X-ray free-electron lasers is described. The instrument uses a novel pump–probe scheme to image down to 940 nm spatial resolution with integration times <100 fs and up to 0.48 GHz frame rates to study the rapid dynamics and small-scale features in laser additive manufacturing.

## 1. Introduction

Metal additive manufacturing (AM) is revolutionizing the fabrication of metallic parts. Laser powder bed fusion (LPBF) in particular uses a scanning infrared (IR) laser for the rapid melting and solidification of regions of a powder bed to form metallic parts. LPBF is an increasingly influential and revolutionary technology with importance to aerospace, bio­medical and defense industries. The additive nature of the manufacturing technique mitigates many tooling constraints of conventional manufacturing to obtain parts of near-arbitrary geometry. This enables increasingly complex and consolidated parts with improved performance and efficiency, and shorter turnaround times in prototyping and design (DebRoy *et al.*, 2018 ▸). However, the variability in microstructural and mechanical performance resulting from rapid dynamic melting/solidification under high-power laser scanning is the quintessential limitation to adopting AM technologies in industrial applications (DebRoy *et al.*, 2018 ▸; Panwisawas *et al.*, 2020 ▸). Thus, there is a need for and use of increasingly sophisticated technologies, from high-speed synchrotron radiographic imaging to advanced computer modeling, to analyze how these microstructures, defects and performance variability arise and can be mitigated (Ioannidou *et al.*, 2022 ▸).

Today, high-speed *in situ* characterization techniques enabled by synchrotrons such as time-resolved X-ray radiography and diffraction have revolutionized our understanding of defect mechanisms, melt-pool fluid flows and the underlying phenomenology of this complex and coupled multiphysics system (Zhao *et al.*, 2017 ▸; Parab *et al.*, 2018 ▸; Huang *et al.*, 2022 ▸; Hojjatzadeh *et al.*, 2019 ▸; Wang *et al.*, 2022 ▸; Gan *et al.*, 2021 ▸; Clark *et al.*, 2020 ▸; Qu *et al.*, 2022 ▸; Guo *et al.*, 2020 ▸; Bitharas *et al.*, 2022 ▸). These synchrotron experiments have explored and directly quantified:

(i) The (in)stability criterion of keyholes (or vapor wells created by metal vaporization under the high-intensity laser) in relation to laser parameters (Gan *et al.*, 2021 ▸; Bitharas *et al.*, 2022 ▸).

(ii) Laser absorption efficiency in relation to keyhole depth (Simonds *et al.*, 2021 ▸).

(iii) Mechanisms of pore formation, incorporation, redistribution and annihilation (Hojjatzadeh *et al.*, 2019 ▸; Huang *et al.*, 2022 ▸; Wang *et al.*, 2022 ▸).

(iv) Generation of the vapor plume from the keyhole and the resulting spatter (Qu *et al.*, 2022 ▸; Bitharas *et al.*, 2022 ▸).

(v) Subsurface fluid flows resulting in pore motion, mass or heat transport and defect mechanics (Guo *et al.*, 2020 ▸).

Most high-speed *in situ* imaging of LPBF has been confined to radiography due to its simplicity, robustness to polychromatic illumination, and tunable contrast mechanisms. The contrast can be tuned between attenuation-based and phase-contrast enhanced by varying the propagation distance to the detector. Polychromatic illumination is often necessary to balance the flux-starved nature of high-speed imaging through metals at synchrotrons. The lateral spatial resolution of radiography is bounded by spatial coherence (source size), detector point-spread function (PSF) or blur kernel, and practical limitations of the Fresnel number not being too small for phase retrieval (if performed) (Diemoz *et al.*, 2012 ▸).

The ‘pixel resolution’ or effective pixel size is commonly reported in the range of ∼2–10 µm (Ioannidou *et al.*, 2022 ▸). This metric assumes a pixel-limited resolution, which would give a lateral spatial resolution ∼4–20 µm per resolvable line pair. In practice, however, the scintillator and other PSFs couple with motion blur to degrade the spatial resolution further beyond the pixel size. Since the resolution of a scintillator-based detector is rarely better than 1 µm (Martin & Koch, 2006 ▸) and the X-rays used for imaging are usually collimated, the above quoted pixel-limited resolution is often a reasonable assumption. Theoretically, quantitative phase retrieval from propagation-based phase contrast imaging (PBI or PCI in the literature) can improve spatial resolution in certain cases (Gao & Cao, 2021 ▸; Gao & Yuan, 2022 ▸), but this is complicated by the source bandwidth and sample complexity and is rarely performed in LPBF studies. Because phase retrieval algorithms used to recover spatial resolution from PCI are rarely used by the LPBF community, spatial resolution is being lost during propagation. Typically, PCI in LPBF studies is used only for qualitative enhancement of edge features, which is a powerful tool in its own right.

When considered as a quasi-continuous X-ray source, the temporal resolution of imaging with a synchrotron is described by the frame rate of the camera used to measure dynamics (Parab *et al.*, 2018 ▸; Tao, 2022 ▸). Currently, the Shimadzu HPV-X2 is the highest frame rate detector used in the LPBF community, with frame rates up to 6.5 MHz, though the frame rates in most LPBF studies range between 1–40 µs (25 kHz – 1 MHz) (Ioannidou *et al.*, 2022 ▸). Exposure times in most synchrotron imaging of dynamics are usually set to the entire interframe spacing due to the flux-limited nature of high-speed imaging through metals; as such, the frame rate and velocity of the image features define the amount of motion blur that appears in the images. However, the exposure time can be dramatically reduced to ∼100 ps duration in certain cases when the pulse trains of the ‘quasi-continuous’ synchrotron source are synchronized with the detector, allowing the images to detect individual pulses at very high frame rates (Parab *et al.*, 2018 ▸). When the dynamics proceed faster than these detector/source limits, pump–probe experiments can be performed in which a slow (often single frame) detector probes the phenomena of interest with some delay Δ from a well defined *t*_0_ initiated by a ‘pump’ (in this case the AM laser). By varying the pump–probe delay over many acquisitions, one can observe faster dynamics than are accessible to high-speed detectors (Alonso-Mori *et al.*, 2015 ▸). For reversible processes, a pump–probe imaging experiment is akin to artificially constructing an arbitrarily fast detector, but for the highly stochastic LPBF phenomena, pump–probe experiments may only describe the statistical evolution of the phenomena over decades of timescales (Hodge *et al.*, 2022 ▸). Due to the complexity of this statistical ‘movie’ and the largely commensurate timescales of LPBF with available fast detectors, pump–probe style measurements are rarely performed.

High-speed LPBF imaging studies have not yet been adapted to the newest X-ray sources, X-ray free-electron lasers (XFELs). High-energy XFELs have emerged in recent years that offer dramatically improved coherence, with a natively small bandwidth Δ*E*/*E* ≃ 10^−3^, a much shorter pulse duration of ∼10 fs and orders of magnitude greater brightness ∼10^12^ photons pulse^−1^ compared to synchrotrons (∼10^9^ photons pulse^−1^) (Alonso-Mori *et al.*, 2015 ▸; Bostedt *et al.*, 2016 ▸; Lehmkühler *et al.*, 2021 ▸; Shen *et al.*, 2007 ▸; Parab *et al.*, 2018 ▸).

In this work, we introduce experiments performed in 2023 on the X-ray Correlation Spectroscopy (XCS) end station of the Linac Coherent Light Source (LCLS) XFEL which use the enhanced XFEL beam attributes to improve spatial and temporal resolution for *operando* LPBF imaging studies beyond what has previously been demonstrated in the LPBF community. We present transmission X-ray microscopy (TXM) with effective pixel sizes down to 206 nm (0.9 µm line-pair resolution) with simultaneous X-ray diffraction (XRD) to validate the material phase. Using the novel multi-pulse train mode available at the LCLS and advanced direct detectors, we further demonstrate up to 2.1 ns interframe spacing (0.48 GHz equivalent). In the text below we briefly introduce our microscope and the trade offs in its design, as well as image processing, before summarizing its resolution and initial results. For a full description of our sample environment, controls integration, photometric calculations and resolution metrics, we refer the reader to the supporting information. Our aim in this work is to demonstrate XFEL-enabled techniques for the LPBF community and to observe the dynamics of this complex multiphysics system on length- and timescales that have previously been inaccessible.

## 2. Experimental

**Figure 1:**
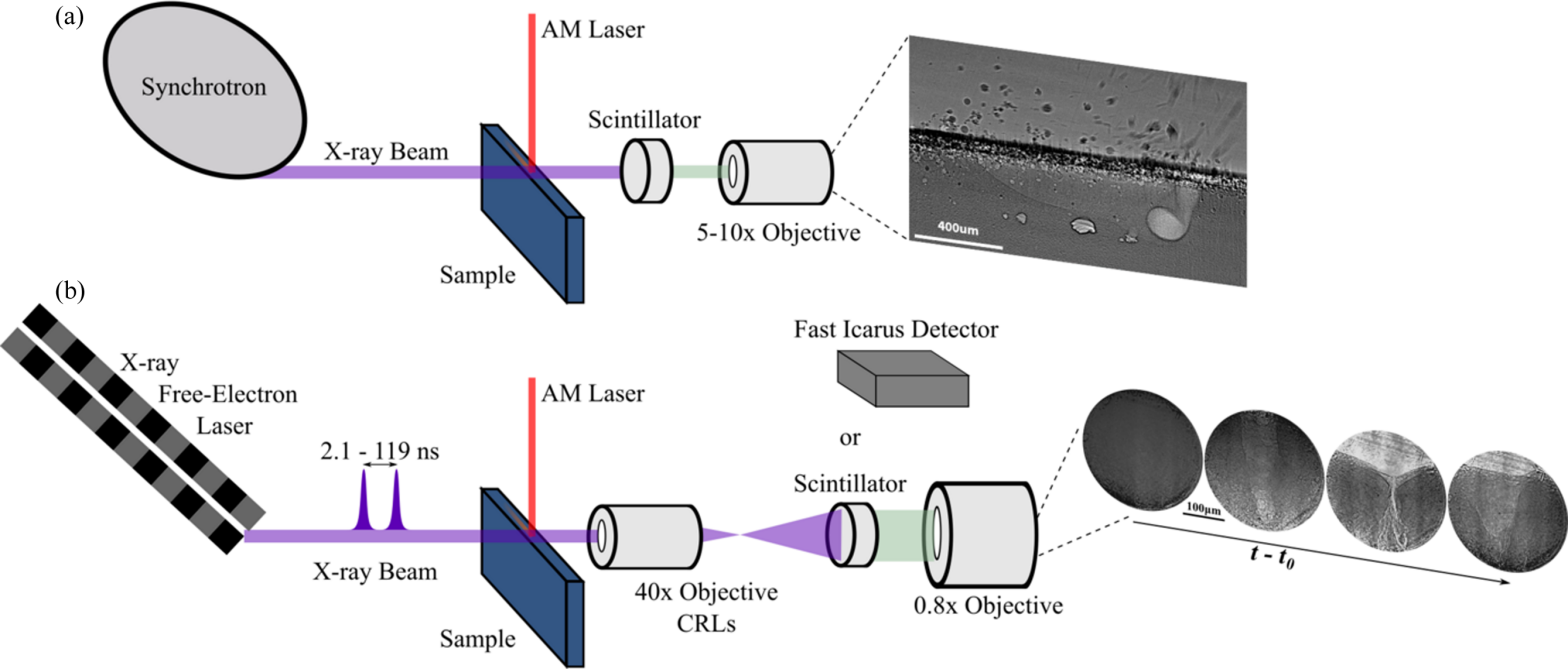
Comparison between (*a*) traditional high-speed radiography at a synchrotron and (*b*) TXM at an XFEL. Synchrotron radiographs of aluminium are shown in (*a*) from our unpublished work.

As shown in Figs. 1 ▸ and 2 ▸, the experimental geometry consists of the microscope, the sample at the laser/X-ray interaction point, the laser optics supplying the beam to the sample, and the timing integration between the laser and X-rays to ensure temporal overlap. We discuss the considerations for each of these experimental components in the sections below.

### 2.1. Microscope design considerations

We used a novel two-pulse accelerator mode at the LCLS in conjunction with the Icarus (or UXI) detector to obtain correlated two-frame ‘movies’ of dynamics in the range from 0.48 GHz to 8.4 MHz. With this approach, our results were able to collect correlated image pairs that span a range of ultrafast phenomena beyond the reach of conventional fast detectors and single-pulse images at synchrotrons (Tao, 2022 ▸). Beyond the inter-frame times, we also used pump–probe timing strategies to measure image pairs over timescales from the initial melt-pool formation through its entire millisecond lifetime, allowing us to obtain uncorrelated statistical ‘movies’ of dynamics spanning nanosecond to millisecond timescales.

We selected TXM to improve the spatial resolution beyond radiography by magnifying the X-ray image before detection (see the supporting information for a full discussion of the nuances of spatial resolution). While achromatic X-ray ‘lenses’ such as Kirkpatrick–Baez (KB) mirrors exist and could be used to perform TXM with less coherent sources, the challenges of fabricating, aligning and maintaining them cause them to be used more commonly in micro- and nano-focusing applications, and relatively few beamlines use them for full-field TXM (Cotte *et al.*, 2018 ▸). Fresnel zone plates are usually used to obtain very high magnification X-ray images, but they are extremely difficult to fabricate for hard X-ray applications and are highly chromatic (Cotte *et al.*, 2018 ▸). We used compound refractive lenses (CRLs) as our objective lenses in this work due to the availability of high-quality CRLs and their simplicity of operation (Cotte *et al.*, 2018 ▸). While CRLs are also chromatic lenses, the temporal coherence (narrow bandwidth) of the LCLS mitigates the CRLs’ chromatic aberrations without requiring a monochromator that also sacrifices beam fluence.

However, one challenge of TXM in the imaging condition is that the contrast mechanism is entirely absorption-based, described by the Beer–Lambert law for a continuously varying medium, integrated over the source spectrum (approximately a delta function in the present analysis) (Diemoz *et al.*, 2012 ▸). The result is that minimal phase contrast exists to offer edge enhancement, and the contrast sensitivity to small features commensurate with the lateral resolution is limited by the sample thickness, source flux and detector bit depth. For this initial demonstration experiment of LPBF imaging at XFELs, magnified phase-sensitive imaging techniques were not implemented, but they could be added in future experiments to modulate the contrast mechanism.

The final challenge of TXM is the trade off between resolution (assuming it is pixel-limited), field of view (FOV), sample thickness and photon energy. Conventional radiography also faces a balance between FOV and resolution set by the number of pixels in the detector (neglecting other issues of blurring). Since TXM introduces an aperture, our FOV is further constrained to at most this aperture size. At higher photon energies, CRLs have less magnifying power, though more X-rays are transmitted through the sample. Thick samples reduce the contrast sensitivity to thin features (along the propagation path), adding to the requirement for high photon energies which boost X-ray transmission and the signal-to-noise ratio; this does, however, lower the lateral spatial resolution. For these reasons, we selected our target magnification to access sub-micrometre effective pixel sizes based on the available detectors and imaging optics. Our selected magnification and the maximum possible microscope length (based on the hutch size) constrain the photon energy, which further constrains the maximum sample thickness possible to achieve a sufficient signal-to-noise ratio for a single-shot image acquisition.

### 2.2. LCLS microscope

In this work, we used a stack of 50 beryllium lenslets with 1.1 mm spacing, 50 µm radius of curvature and 300 µm aperture (note CRLs are parabolic in profile). Using the ray transfer matrix formalism described by Simons *et al.* (2017 ▸) to describe the focusing properties of CRLs, we designed our TXM setup to operate with 11 keV X-rays. Fig. 1 ▸ compares the configurations for synchrotron radiography and TXM at XFELs with example results. Our microscope was 8.264 m in length with an effective focal length of 143 mm, a working distance of 148 mm, an expected magnification of 37.64× and a measured magnification of 39.4×. To minimize X-ray attenuation from the beam propagating through air, we propagated the beam through a vacuum-filled beam tube for the longest distances of beam propagation. In addition to this CRL, a diamond aberration-corrective phase plate was used to correct and improve the CRLs’ resolution and imaging quality (Seiboth *et al.*, 2017 ▸). Because of this aberration correction, the spatial resolution limit of our microscope was limited by the detectors’ effective pixel size and our source bandwidth.

Two detectors were used in this work. The Icarus detector (also described as the UXI detector) produced by Sandia National Laboratories is used to image ultrahigh speed phenomena such as shockwaves and inertial confinement fusion (Dayton *et al.*, 2016 ▸; Hodge *et al.*, 2022 ▸; Looker *et al.*, 2020 ▸). As a four-frame burst-mode X-ray direct detector with single-photon sensitivity and 25 µm pixels in a 500 × 1000 grid, the interframe spacing can be varied between 1.6–20 ns (Hart *et al.*, 2019 ▸; Hurd *et al.*, 2018 ▸). The internal clock was modified to augment this range of interframe spacings up to 55 ns for this experiment, allowing the detector to image the two pulses of the LCLS pulse train directly and separately (Hart *et al.*, 2019 ▸). The other detector is an Andor Zyla 4.2 sCMOS camera imaging a 50 µm thick 25 mm diameter LuAG:Ce scintillator placed at the TXM image plane, using a 0.8× zoom lens (Navitar Zoom 7000) with NA = 0.03 and a 45° mirror for X-ray to optical conversion. The 6.5 µm pixel size with 2000 × 2000 pixel active area gave a higher spatial resolution but integrates the two XFEL pulses on the imaging detector. The indirect detection scheme was also less efficient, and thus was unable to capture useful images for the lowest-intensity pulse trains.

To evaluate the appropriate magnification, FOV and signal on our detector, we used the *Photometrics* toolbox introduced by Taylor (2025 ▸). See the supporting information for a discussion of the toolbox implementation and results for our experiment.

Simultaneous XRD was measured on an ePix10k-Quad detector adjacent to the sample. This detector integrates both XFEL pulses together with single-photon sensitivity, capturing a 2θ range of 33.3°–99° or *q* = 3.19–8.48 Å^−1^ with dθ = 0.03° angular resolution. In this work, the XRD signal primarily helped to inform whether the sample was in the solid or liquid phase for each measurement. While our experiments in this work did not explore metastable phases formed during solid­ification in LPBF, the simultaneous XRD and TXM could offer direct correlation between phase and structure for future experiments with more complex material systems.

### 2.3. Alignment

Because CRLs are thick lenses, they must be carefully aligned collinear to the X-ray beam. Grazing-incidence reflections from the sides of the CRL tank were used to align the two rotation axes perpendicular to the X-ray beam. Coarse CRL alignment along the *z* axis (*i.e.* the optical axis) is performed from calculations based on the method of Simons *et al.* (2017 ▸). A focal sweep was used to find the focus, varying the CRL position along the *z* axis and imaging a TEM grid. When the grid edges were in the middle of the range associated with best focus (*i.e.* the edges of the grid made the thinnest lines), the TXM was considered in focus. The AM laser was used to drill a crater in the sample, which was used to shift the AM laser alignment to obtain spatial overlap with the X-rays. A photodiode monitoring the AM laser emission was used to validate the X-ray-to-visible laser temporal overlap.

### 2.4. Sample environment

**Figure 2:**
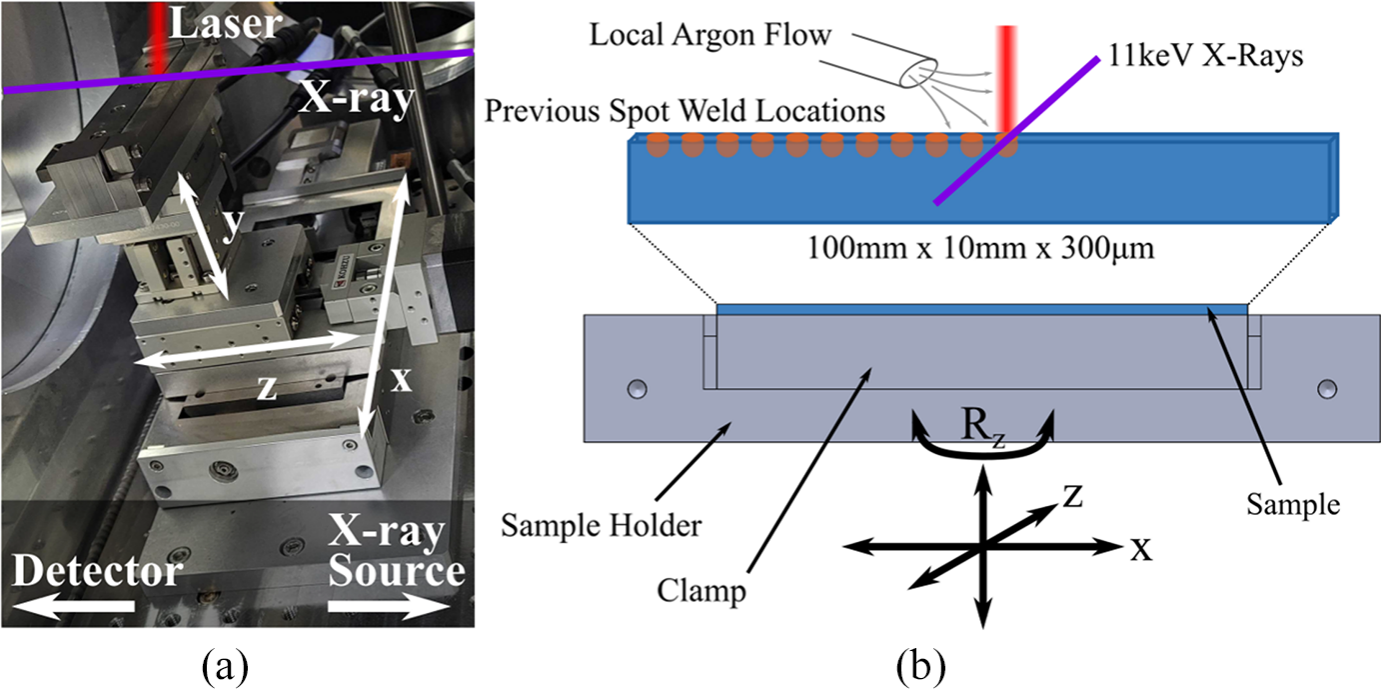
(*a*) Sample geometry, holder and stage stack with rotations to align the sample at (*b*) the interaction point. The sample was moved between spot-weld locations, while the laser and X-ray interaction point remained fixed with a local argon supply.

An nLight AFX1000 1070 nm 1200 W continuous-wave (CW) laser was used as the AM ‘pump’ laser for 3 ms spot welds on aluminium 6061-T6 alloy samples of 300 µm thickness. The laser interaction point with the samples was placed under argon shielding gas at 2 L min^−1^ flow rate instead of completely purging the environment, as shown in Fig. 2 ▸. There is precedent for the use of shielding gas and a local argon environment in place of a complete argon environment in LPBF studies (Scipioni Bertoli *et al.*, 2017 ▸), but we note that there was noticeable oxidation at the laser spots studied in these experiments. The laser was triggered to turn on at a time relative to the XFEL trigger with pump–probe delays Δ between 0 and 5 ms to track laser melting and solidification. Laser powers were varied up to 800 W.

### 2.5. Image processing

The raw images collected in this work exhibited a few types of imaging artifacts. Shot-to-shot fluctuation of the XFEL beam arises from the self-amplified spontaneous emission (SASE) process used to create the pulses, which generates an uneven and variable background illumination for imaging experiments. Fringing and partial reflections result from debris and upstream XFEL optics outside of the TXM imaging condition. Some of these features jitter spatially with the SASE fluctuations to contribute imaging artifacts, but do not contain any information about the sample. Burns or in­homo­geneities in the CRLs may also introduce artifacts when not properly accounted for by the aberration-corrective phase plates.

The Zyla images were post-processed by dark-subtracting both the raw and flat images, computing an average flat-field image, and dividing the dark-subtracted raw image by this average dark-subtracted flat-field image. This method performed quite well and advanced techniques based on principal component analysis (Birnsteinova *et al.*, 2023 ▸) or inverse problem solving with priors (Venkatakrishnan *et al.*, 2013 ▸) were not needed. All images were then normalized in intensity by their 99% percentile to obtain consistent illumination. In the future, normalizing the image intensity by the pulse energies will give a more representative estimate of sample transmission, allowing the Beer–Lambert law and other calculations to be possible.

For the Icarus images, the background profiles were more complex because the image spans two detector panels (Dayton *et al.*, 2016 ▸; Claus *et al.*, 2017 ▸), with column-wise intensity variations in the dark. For the Icarus signals, a special process was completed for both the raw images and the flat-field images (described below), before the images were flattened in the same manner as the Zyla. For the additional procedure, the ratio of the image signal to the averaged dark signal (masking out the region where the TXM image illuminated the detector) was used to rescale the dark image column-wise prior to subtraction from the image. After the above procedure, the two panels were then compared in the un-illuminated pixels to calculate an offset between the two detector panels, which was then used to align the intensity of the panels at their interface.

## 3. Results

We begin by introducing our measurements for spatial and temporal resolution and quantifying these attributes explicitly. We then provide representative images showing the data collected with our TXM and XRD configuration, and use them to demonstrate the typical findings achievable with our *operando* LPBF at the LCLS.

### 3.1. Spatial resolution

Spatial resolution and magnification were characterized by imaging a Siemens resolution star (XRESO-50HC). A 2000 mesh TEM grid gives direct calibration of the FOV, including a calibration of the aberration corrective optics – which reduced the spherical aberrations native to the parabolic shape of the CRLs. Our spatial resolution for holographic images (without the CRLs) was calibrated with a 400 mesh copper TEM grid, discussed in the supporting information.

**Figure 3:**
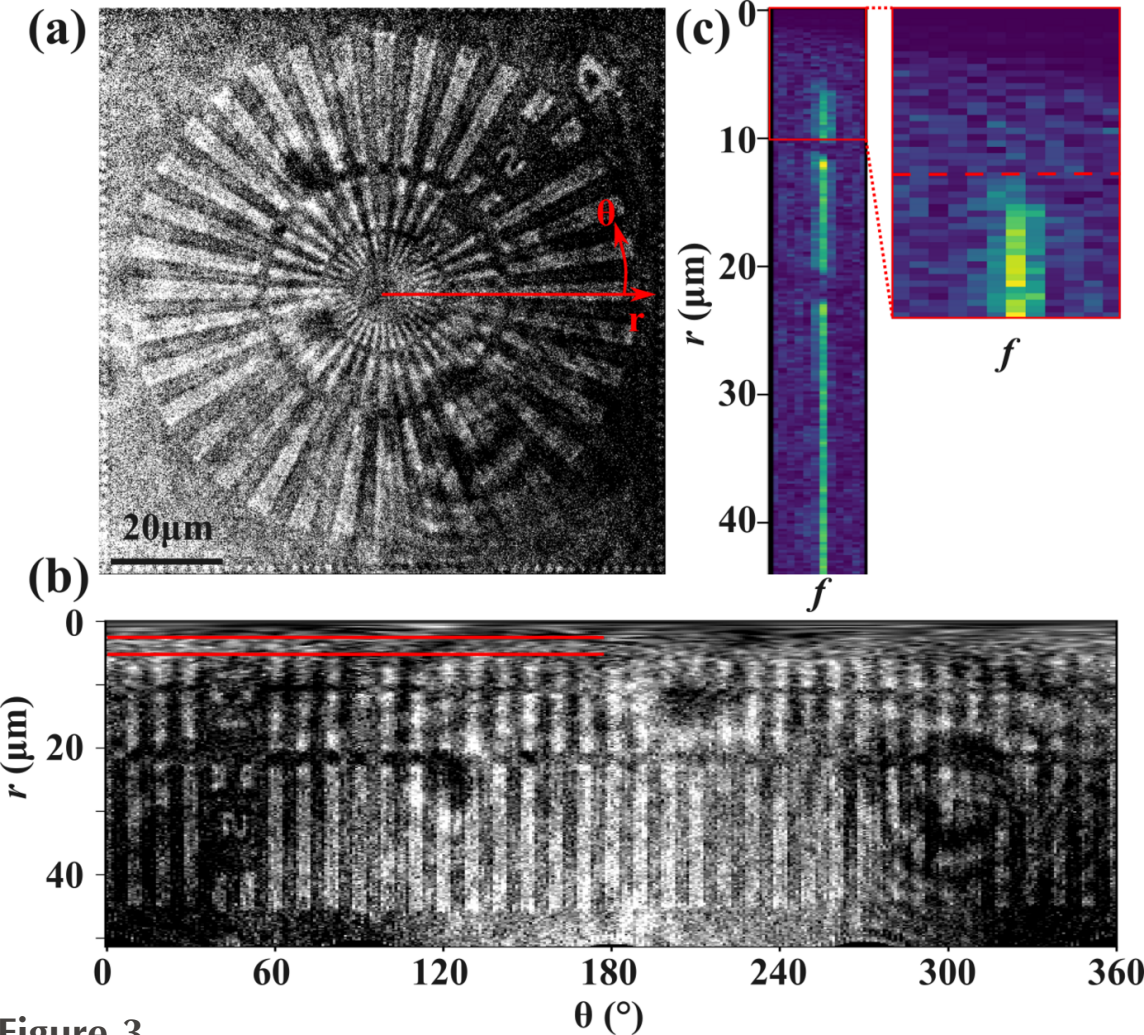
(*a*) A 90 µm Siemens resolution star imaged using the Zyla detector. (*b*) The polar transform of this image, with the two red lines marking the two-pixel resolution limit (0.4 µm) and the actual resolution at which the line pairs are no longer resolvable (0.9 µm). (*c*) A subset of the Fourier transform of the polar transformed image corresponding to the Siemens star spokes, with an inset and dashed red line marking the point at which the star is no longer resolvable.

On the Siemens resolution star shown in Fig. 3 ▸, three concentric rings are resolvable, indicating a resolution of at least 1 µm per line pair. To determine the resolution quantitatively, this circular Siemens star was polar transformed into radial and azimuthal coordinates, and then the Fourier transform of this image across the azimuthal coordinate was taken. The peak corresponding to the lines of the Siemens star is shown in Fig. 3 ▸(*c*) as a function of the radial coordinate, and the point at which this peak can no longer be resolved gives the radius of the minimum-sized circle that can resolve the Siemens star. Dividing the circumference of that circle by the number of lines in the star (36) gives the smallest resolvable line-pair distance. From the known 90 µm diameter of the Siemens star, the effective pixel size and magnification are obtained.

From this analysis, our microscope using the Zyla indirect detector had an effective pixel size of 206 nm pixel^−1^ with a spatial resolution of 940 nm and 31.55× total magnification (39.43× X-ray and 0.8× optical, after the scintillator). With the Icarus direct detector, the microscope had a resolution of 1.3 µm with 39.4× magnification and 635 nm pixel^−1^ effective pixel size. These values are consistent with a detector-limited resolution based on the pixel size of the Icarus camera, but suggest that chromatic and other aberrations as well as scintillator blurring reduced the resolution for the indirect detector. Approximately 0.4 µm line-pair resolution would have been expected using the indirect detector with a pixel-limited resolution, but the actual resolution was more than double this estimate. Details of all magnifications, effective pixel sizes and resolution for the two detectors is given in Table 1 ▸.

Using the formalism of Simons *et al.* (2017 ▸), we expect the FWHM of the PSF of the CRLs due to their aperture to be approximately 0.2 µm according to equation (31) of that work. The chromatic aberration from equation (39) of Simons *et al.* (2017 ▸) is then expected to be ∼4 µm per %bandwidth, which equates to about 1.3 µm FWHM of chromatic aberration with normal XFEL operation in the hard X-ray regime (Δ*E*/*E* = 3 × 10^−3^). However, the actual PSF due to chromatic aberration can be four-fold or more reduced from this value if a full wave-optical treatment of CRL performance is considered (Pedersen *et al.*, 2018 ▸). Further, the resolution from the scintillator using indirect detection is about 6 µm at the scintillator or 0.2 µm at the sample (Martin & Koch, 2006 ▸). The spherical aberration from the CRLs was not measured, but is expected to be minimal after the aberration-correcting phase plates (Seiboth *et al.*, 2017 ▸). All of these PSFs are convolved with the original image, adding in quadrature to obtain the measured resolution, suggesting that chromatic aberrations due to the SASE bandwidth comprise the majority of the resolution limits of our microscope. In high-charge modes with appropriate monochromation, or possibly using a seeded XFEL operation, it is expected that most of these chromatic effects using CRLs could be mitigated with less than a one-order reduction in signal. For reference, the two red lines in Fig. 3 ▸(*b*) indicate, respectively, the pixel-limited and measured resolution limits of the Siemens star.

For comparison, the CRL was removed and the transmitted beam was imaged with propagation-based phase contrast (well into the holography regime). Since the XFEL beam had very little divergence, the 10× magnification was thus all optical (*i.e.* 10× objective lens attached to Andor Zyla) using the Zyla indirect detector, with a 0.65 µm pixel^−1^ effective pixel size and approximately 30 µm resolution without phase retrieval, using a Fresnel number equal to unity to define the discernible feature size. With the removal of the aperture, the FOV was limited only by the X-ray beam width to approximately 1.2 mm.

### 3.2. Temporal resolution

The temporal resolution in this work is defined by the integration time of the images, the frame rate of our two-pulse mode operation and the jitter of our pump–probe delays.

The pulse duration of the configuration we used at LCLS is <100 fs, over which atomic motion is negligible, resulting in completely motion blur free imaging (Bionta *et al.*, 2014 ▸; Bostedt *et al.*, 2016 ▸; Hwu & Margaritondo, 2021 ▸).

Our experiments were conducted in two-pulse mode (Decker *et al.*, 2022 ▸). The XFEL pulses were separated by 2.1, 21 and 119 ns, respectively, measured by fast diode traces. The Icarus detector could resolve these two pulses independently and, with its measured 1.3 µm resolution, the minimum resolvable velocities projected onto the imaging plane are listed in Table 2 ▸. The maximum measurable velocities are calculated assuming a feature could be correlated over 20 resolutions (25 µm or 40 pixels). The Zyla detector integrates these pulses together, thus experiencing motion blur for very high velocities, and is not used to estimate feature motion. For comparison, the velocities of common physical features in AM are reported in Table 3 ▸ (Guo *et al.*, 2020 ▸; Qu *et al.*, 2022 ▸; Bitharas *et al.*, 2022 ▸; Yin *et al.*, 2020 ▸; Chen *et al.*, 2024 ▸; Alexeev *et al.*, 2020 ▸; Trachenko *et al.*, 2020 ▸). Our microscope was best suited for probing cracking, fracture and shockwaves, if present.

The r.m.s. timing jitter of the XFEL in its two-pulse mode is ∼10 fs (Decker *et al.*, 2022 ▸) and the FWHM jitter of the trigger pulse is less than ∼250 fs (Bionta *et al.*, 2014 ▸; Bostedt *et al.*, 2016 ▸). Both of these are many orders of magnitude less than the AM laser jitter of approximately 30 µs. The dead time between the trigger and laser response was found by fast photodiode traces to be trimodal at about 85 µs, with each sub-peak demonstrating about 3 µs r.m.s. jitter and 5.5 µs rise time. On the millisecond scale of laser melting and solidification, this jitter is negligible.

### 3.3. Representative LPBF results

**Figure 4:**
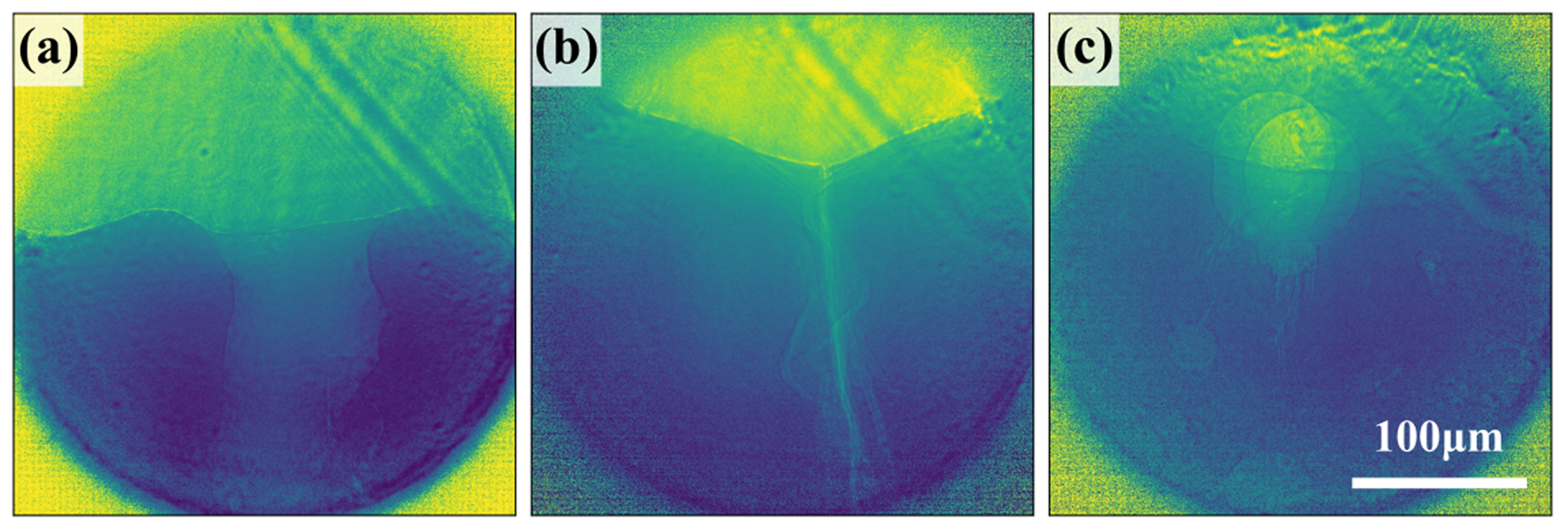
TXM images of (*a*) a keyhole, (*b*) hot cracking and (*c*) partially solidified pores, as measured on the Zyla detector.

**Figure 5:**
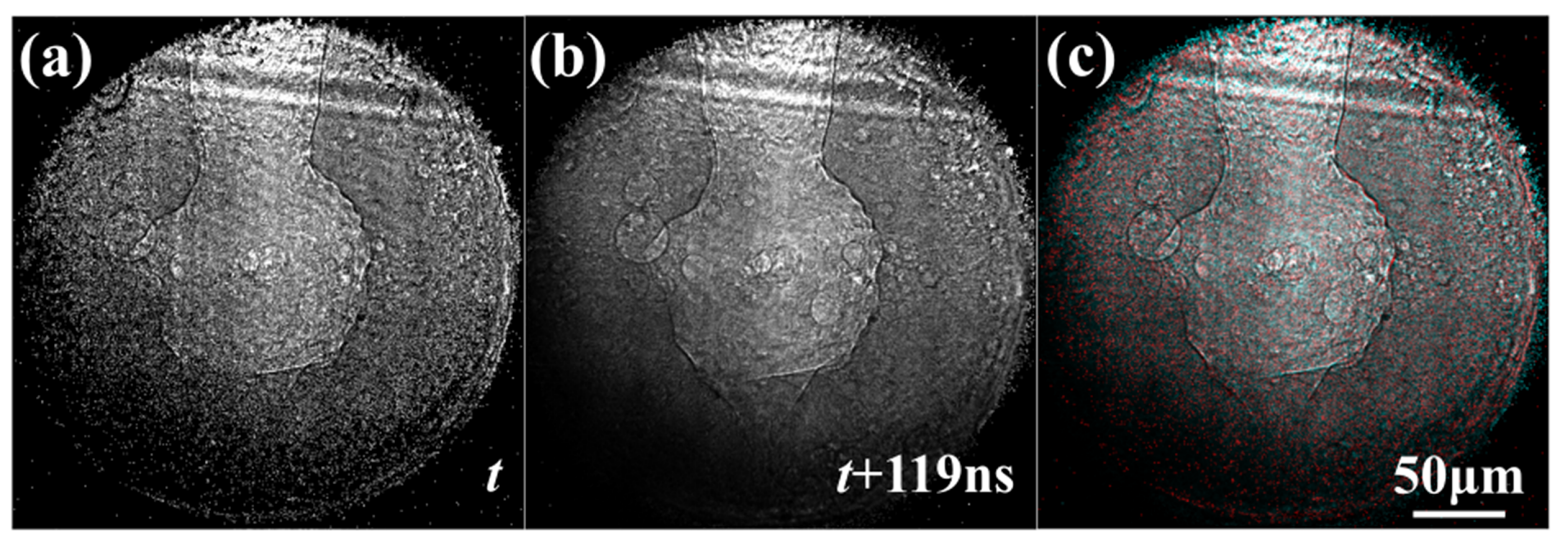
(*a*) A keyhole generated at 700 W laser power after 0.45 ms and (*b*) 119 ns after the first frame, as measured on the Icarus detector. Both are overlaid and color-coded in panel (*c*), where red signal indicates more signal on the first frame and cyan signal indicates more on the second. Two-pixel feature motion equivalent to 10.7 m s^−1^ velocity.

The images measured by our microscope were able to resolve the most commonly observed features of LPBF: keyholes, solidification cracking, solid–liquid interface and pores. Examples are shown in Fig. 4 ▸. Our novel microscope is best suited to observing the high-velocity physics of laser melting and solidification present in LPBF. With the velocity ranges probed in our two-pulse trains, our microscope is most sensitive to very rapid keyhole motion, cracking and even shockwaves. However, no obvious motion was observed in any of our experimental results. Shockwaves were not visible, and hot cracking, keyhole motion and pore motion all proceeded at velocities below the detection limits of this microscope, ∼10 m s^−1^. Fig. 5 ▸ shows the first and second probe images captured on the Icarus detector for a keyholing laser condition, as well as a composite image that is red where signal intensity was lost and blue where it was gained. Signal variability and imperfect flattening make it difficult to discern feature velocities absolutely near the lower detection limit of our microscope. Due to speed-of-sound limitations, no macroscale physics would be expected to move faster than the detection limit of our microscope. While not a disproof, these observations do not support the supposition of shockwaves induced in LPBF.

The findings from these images were primarily pore accumulation and growth from the nano- to the micrometre scale over successive laser (re)melting and solidification at the same location. Over time, smaller pores were observed to accumulate at the boundary defining the maximum melt-pool extent, sometimes consolidating to form larger pores. This indicates that bubble escape mechanisms commonly cited in the literature were not active in these successive remelting conditions. A thorough discussion of these results is given by Fang *et al.* (2025 ▸).

**Figure 6:**
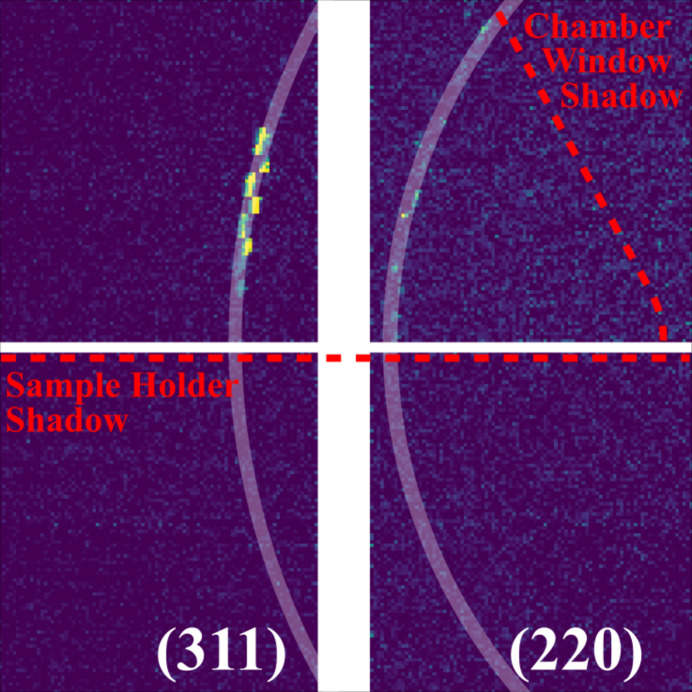
*In situ* XRD pattern captured on the ePix10k-Quad detector in a solidified sample.

Our simultaneous XRD measurements confirmed our imaging interpretation by TXM that the Al6061 alloy melted in the FOV of the XFEL during laser scans. The (311) and (220) peaks visible in the solid sample decreased in intensity when probed with a small melt pool and disappeared when completely melted. An XRD pattern measured for the solid phase is shown in Fig. 6 ▸ with overlays of the illuminated region and diffraction patterns. Signal is low because the large spot size necessary for TXM was not optimized for XRD (*i.e.* smaller spot size for higher diffraction resolution).

**Figure 7:**
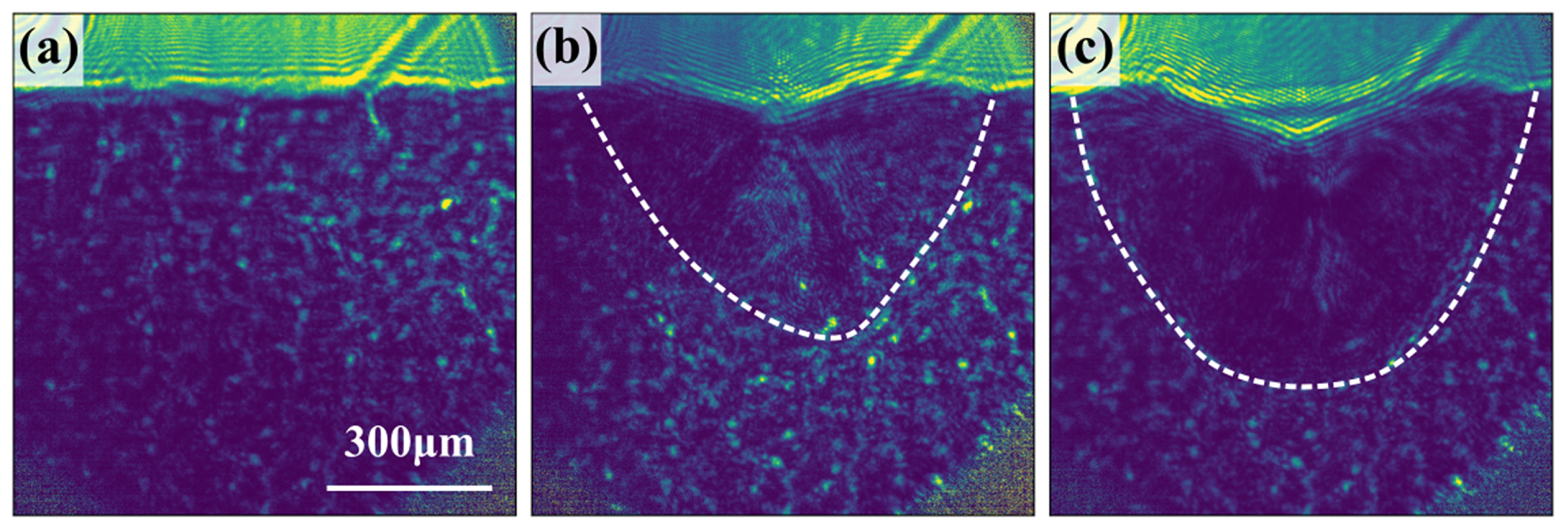
Holographic imaging of the melt pool with increasing time steps, (*a*) 0 ms, (*b*) 0.75 ms and (*c*) 0.9 ms, at 700 W of power measured on the Zyla detector.

For comparison, we also show the holographic images measured during the laser melting process with a wide FOV in Fig. 7 ▸. These show the wide extent of the melt front beyond the ∼300 µm FOV for TXM, but the interference and fringing prohibit more detailed analysis. In the present study, conventional holographic reconstruction was not achievable because the imaging signal necessary for reconstruction extends beyond the edges of the measured region. More advanced reconstruction techniques will be explored for future interpretation of these results.

## 4. Discussion

Our TXM microscope for LPBF demonstrates motion blur free images with the highest spatial and temporal resolution used to date by any *in situ*X-ray technique for AM. Our microscope was optimized to probe dynamics at velocities not typically observed in AM, including the nanosecond dynamics and nanoscale porosity of the melt pool.

As the first time an *operando* LPBF experiment has ever been implemented at an XFEL, we employed a local argon environment, which resulted in significant oxidation, and a stationary laser that focused our study on spot-welding conditions. Our future studies will advance the present approach by implementing laser scanning and using an argon-purged enclosure to probe conditions that more closely resemble traditional *operando* LPBF studies. With impending upgrades to various XFEL facilities world-wide to megahertz timescales and the increasing availability of fast detectors, our TXM results will become feasible over temporal ranges that offer multi-frame videos of the LPBF dynamics.

The primary limitation of this microscope (TXM) relative to synchrotron radiography is the decreased FOV (∼300 µm) imposed by the aperture of the CRLs. Without the context of the full melt pool and surrounding substrate, the melt-pool size cannot be determined and feature dynamics outside the FOV are unknown. Work could be done to enlarge the CRL apertures, but this places limits on their magnifying power and resolution, increasing the length of the microscope or decreasing its performance. Future studies may benefit from exploring alternate focusing optics for the TXM geometry, or aperture-free magnification techniques.

## 5. Conclusion

In this work, we have demonstrated the first *operando* TXM of laser melting at an XFEL with 0.9 µm spatial resolution and temporal resolution variable between 2.1 and 119 ns over two pulses.X-ray diffraction over a limited *q* range was implemented concurrently with TXM to track the material phase within the limited FOV. For comparison, we have also reported holography over an 8.2 m propagation distance.

The advantage of this microscope in improving the spatial resolution of *operando* imaging for LPBF and related fields, however, is clear. Due to the coherence, bandwidth and brilliance of XFELs, well developed imaging techniques which have been implemented for other fields are now viable on the timescales necessary to probe the fluid physics, chemistry, solidification and microstructural dynamics of LPBF in ways never before accessible. Our work implementing TXM with attenuation-based contrast is just the simplest of these advancements to be made.

## Supplementary Material

Supporting information. DOI: 10.1107/S1600577525001675/tv5071sup1.pdf

## Figures and Tables

**Table 1:** Magnifications and line-pair resolutions

Field of view	298 µm
X-ray magnification	39.4×
Optical magnification	0.8×
Zyla resolution	0.9 µm
Zyla effective pixel size	206 nm pixel^−1^
Icarus resolution	1.3 µm
Icarus effective pixel size	635 nm pixel^−1^

**Table 2:** Measurable velocities on Icarus

Pulse separation (ns)	Minimum velocity (m s^−1^)	Maximum velocity (m s^−1^)
2.1	600	12000
21	60	1210
119	11	210

**Table 3:** Velocity of AM phenomena

Phenomenon	Velocity range (m s^−1^)
Solidification and melting	0.1–1
Pore motion	0.1–4
Keyhole motion	1–10
Spatter	1–10
Tearing	1–10
Cracking	10–100
Vapor plume	100–1000
Fracture	100–1000
Shockwaves	1000–10000
