# Supplementary material for: High-resolution in situ characterization of laser powder bed fusion via transmission X-ray microscopy at X-ray free-electron lasers
Source: J Synchrotron Radiat. 2025 Apr 1;32(Pt 3):524–33. doi: 10.1107/S1600577525001675 (PMC12067346; doi:10.1107/S1600577525001675)
Supplement: Supplementary file 1 [file s-32-00524-sup1.pdf]

JOURNAL OF SYNCHROTRON RADIATION

Volume 32 (2025)

Supporting information for article:

High-resolution *in situ* characterization of laser powder bed fusion via transmission X-ray microscopy at X-ray free-electron lasers

Zane Taylor, Tharun Reddy, Lichao Fang, Patrick Oppermann, Patrick L. Kramer, Franz-Josef Decker, Matthew Seaberg, Matthieu Chollet, Tim van Driel, Alex Halavanau, Philip Hart, Matthew Dayton, Frank Seiboth, Wenxin Wang, Carolyn Gee, Abigail Wilson, Rachel Margraf-O'Neal, Gourab Chatterjee, Ying Chen, Ilana J.P. Molesky, Yifan Wang, Sara Irvine, Jade Stanton, Cynthia Melendrez, Kelsey Banta, Silke Nelson, Vivek Thampy, Kento Katagiri, Morten Haubro, Sen Liu, Dayeeta Pal, Lauren Moghimi, Christopher Tassone and Leora Dresselhaus-Marais

Supplemental Information: High-Resolution *In-situ* Characterization of Laser Powder Bed Fusion via Transmission X-ray Microscopy at X-ray Free Electron Lasers

Zane Taylor,[a,b] Tharun Reddy,[a,b] Lichao Fang,[a,b] Patrick Oppermann,[c] Patrick L. Kramer,[c] Franz-Josef Decker,[c] Matthew Seaberg,[c] Matthieu Chollet,[c] Tim van Driel,[c] Alex Halavanau,[c] Philip Hart,[c] Matthew Dayton,[d] Frank Seiboth,[e] Wenxin Wang,[e,f] Carolyn Gee,[c] Abigail Wilson,[c] Rachel Margraf-O'Neal,[a,c,g] Gourab Chatterjee,[c] Ying Chen,[c] Ilana J.P. Molesky,[a,b] Yifan Wang,[a,b] Sara Irvine,[h,b] Jade Stanton,[h,b] Cynthia Melendrez,[c] Kelsey Banta,[c] Silke Nelson,[c] Vivek Thampy,[i] Kento Katagiri,[a,b] Morten Haubro,[j] Sen Liu,[i,k] Dayeeta Pal,[a,b] Lauren Moghimi,[a,b] Christopher Tassone[i] and Leora Dresselhaus-Marais [a,b]*

[a]*Department of Materials Science and Engineering, Stanford University, Stanford, CA 94305, USA,* [b]*SLAC National Accelerator Laboratory, Menlo Park, CA 94025, USA,* [c]*Linac Coherent Light Source, SLAC National Accelerator Laboratory, Menlo Park, CA 94025, USA,* [d]*Advanced hCMOS Systems, Albuquerque NM 87107, USA,* [e]*Center for X-ray and Nano Science CXNS, Deutsches Elektronen-Synchrotron DESY, Notkestr. 85, 22607 Hamburg, Germany,* [f]*European X-ray Free-Electron Laser Facility, Holzkoppel 4, 22869 Schenefeld, Germany,* [g]*Argonne National Laboratory, Lemont, IL 60439, USA,* [h]*Department of Applied Physics, Stanford University, Stanford, CA 94305, USA,* [i]*Stanford Synchrotron Radiation Lightsource, SLAC National Accelerator Laboratory, Menlo Park, CA 94025, USA,* [j]*Department*

of Physics, Technical University of Denmark, Kongens Lyngby, Denmark, and
[k]*Department of Mechanical Engineering, University of Louisiana at Lafayette, Lafayette, LA 70503, USA. E-mail: leoradm@stanford.edu*

1. AM Laser Integration

The AM laser used in this experiment was an nLight AFX1000 1070 nm 1200W system acting as the "pump" source to initiate the laser-melting dynamics. The optical components to collimate, focus, and steer the laser beam were assembled on a breadboard above the sample environment, and reflected downward into the sample chamber. The nLight fiber laser had an output optical fiber fed directly into the nLight collimator then output onto a breadboard. The collimated beam was then fed into our home-built Galilean beam expander with a 1/2" lens (L1) of $f = -25$ mm followed by a 2" $f = +100$ mm lens (L2), for which the distance between these AR-coated lenses was manually adjusted to control the coarse focus of the entire optical system. After the beam expander, the beam passed through an AR-coated 2" $f = +1000$ mm focusing lens (L3) mounted onto an x-y-z stack of translation stages to provide fine adjustment of the laser spot size and position at the sample.

A calcium fluoride window (3 mm thick) reflected a small portion of the beam onto a 2.3-ns fast photodiode (PD) to monitor laser timing features and power. In particular, the delay time between the "laser-on" trigger and power output from the laser (a.k.a. the "laser-on-time") and the rise-time of the leading edge of the pulse are important to calibrate the AM experiments at XFELs. The timing precision of the XFEL requires calibration of the AM laser beyond the typical resolution of industrial-grade manufacturing lasers, necessitating on-line monitoring to evaluate the properties of the laser pulse after each experiment. See Fig. 1 for a full schematic of the AM pump

laser's optical path.

The power of the AM laser was measured using a commercial thermal power meter located at the asterisk in Fig. 1(a) and used to calibrate the laser's voltage-control shown in Fig. 2. The photodiode signal used to monitor laser timing was also calibrated using the thermal power meter so that power could be monitored when the power meter was removed during the experiment. The results of the power calibration and a typical laser response profile are shown in Fig. 2(a) and (b), respectively.

2. Controls Integration

The laser was operated in an analog mode. 24 V signals were applied to the hardware control port to trigger laser activation, enabling, emission, gating, and power. All switches were manually but remotely flipped and integrated into the hutch controls system. The interlocks were likewise integrated into the hutch laser safety system (LSS).

Once fully enabled and in the emission state, the laser requires two signals to fire in analog mode: a gate and a power. The gating channel must be active and is a binary switch as the laser's final safety, while the voltage on the power channel must be set to control the wattage of the laser output power. The gating channel was manually and remotely controlled as previously mentioned, but the power output was set by TTL signals produced by the delay generators ubiquitous in XFEL research.

The 4 V TTL signals were sufficient to trigger up to 800 W emission on the nLight laser system, were easily integrated into the hutch controls system, and allowed the laser timing to be controlled relative to the XFEL beam. Delays on this channel set the pump-probe delay of the experiment (to within the laser's jitter time).

TTL signals from delay generators provided the necessary triggers for the detectors, including the Andor Zyla and Icarus which were recorded into the data acquisition

system (DAQ).

3. Accelerator Mode

This experiment leveraged the recently developed 2-pulse operation mode demonstrated previously for accelerator development and shock wave dynamics (Decker *et al.*, 2022; Hodge *et al.*, 2022). These pulses were produced upstream of the photoinjector (where electrons are initially injected into the linear accelerator prior to entering the undulator), wherein two lasers are used to drive photoinjection that were temporally offset by 2.1 – 119 ns. This produced two electron bunches which were accelerated and lased collinearly through the undulator to produce two X-ray pulses spaced by 2.1 – 119 ns in time. A number of modifications must be introduced to the accelerator operation to correct for the interaction of the two electron bunches with each other during their orbit and acceleration. There is an additional loss of XFEL flux as the two pulses are further separated in time due to losses in laser intensity prior to the photoinjector, and lost efficiency in the accelerator. A 6.4 GS/s queued analog-to-digital converter (Abaco Systems FMC134) was used to monitor the pulse train quality.

The novelty of this 2-pulse mode is the ability to resolve correlated GHz scale dynamics with our pump-probe experiments at the LCLS. Most XFELs operate by generating a high-frequency "pulse train" which contains only a small number of pulses, and these pulse trains then operate at a much lower repetition rate. Some XFELs, such as EuXFEL, can generate 2700 pulse, 4.5 MHz pulse trains at a 10 Hz repetition rate (Lehmkühler *et al.*, 2021). LCLS-I previously operated in a 1-pulse train at up to 120 Hz (typically limited by the detector speed). This new 2-pulse operation mode at LCLS allowed us to operate 2 pulse, 0.48 GHz – 8.4 MHz pulse trains at up to 120 Hz.

In practice, available detectors limited the repetition rate to 1 Hz and 15 Hz, which is longer than the pump laser pulse duration of 3 ms and subsequent solidification time of approximately 3 ms. Because of this repetition rate, dynamic correlated "movies" as traditionally captured by high-speed radiography at synchrotrons are only possible within pulse trains, and not between them at XFELs. For this reason, our experiment operated in a pump-probe configuration: the AM or pump laser was turned on, and after some delay, the XFEL 2-pulse pulse train, or probe, imaged the resulting system. Dynamic correlations are only possible between the two pulses of the pulse train and no correlation exists between each pulse train.

4. Experimental Methods

The 100-mm sample was imaged every 2mm before, during, and after the successive AM laser acquisitions. We probed the incipient dynamics, steady-state dynamics, and solidification processes using a "pump-probe" approach outlined in Fig. 3. Our AM laser had a 3-ms pulse duration, which arrived at the sample with every other XFEL pulse to probe before and after each AM laser pulse. The XFEL pulses arrived at a 1–15-Hz repetition rate depending on the detector. Our 2-pulse XFEL probes (fixed inter-pulse delays of 2, 21, and 119 ns) arrived at a set time delay, Δ, after the arrival of the AM laser; the time delays ranged from $\Delta = 10$-ns through 5-ms. In this way, by collecting a statistical number of acquisitions at each value of Δ, our experiment could measure the statistics of the bubble dynamics and crack formation that occurred in each representative decade of timescales in the melt-pool and solidification dynamics. As the first LPBF study at an XFEL and a pump-probe experiment, we chose to keep the laser stationary and study spot-welding dynamics of laser melting and solidification: varying P and Δ.

5. Spatial Resolution

Spatial resolution and magnification were characterized by imaging a Siemens resolution star (XRESO-50HC) and TEM grids (2000 Mesh), respectively. Fig. 4 shows the resolution measurement analysis described in the main text for the Zyla, but for the Icarus detector. On a TEM grid, the resolution may be measured by the number of pixels defining the sharp air-metal interface that defines the the TEM grid. It is worth noting that for a fully periodic TEM-grid structure, images must be collected with the non-periodic edge of the grid in the field of view to avoid mistakenly attributing aliasing artifacts from the Talbot effect (when out of focus) to the in-focus image. The use of a TEM grid offers direct calibration of the field of view, even including any spherical aberrations in the image, based on the number of squares that can be measured along each direction. In our imaging system, the TEM grids also offered calibration of the aberration corrective phase plate – which reduced the spherical aberrations native to the parabolic shape of the CRLs. Our spatial resolution for holography images (without the CRLs) were calibrated with a 400 Mesh copper TEM grid, as shown in the comparison to TXM shown in Fig. 5.

For comparison, the CRL was removed and the transmitted beam was imaged in the holographic regime with propagation-based phase contrast, as shown for a TEM grid in Fig. 5(b). Fig. 5(a) is an image of a 2000 Mesh (12.5μm pitch and 7.5 μm hole) TEM grid while 5(b) is an image of a 400 Mesh (63 μm pitch and 30 μm hole) TEM grid. Since the detectors were not otherwise moved, this far-field imaging results in holography (propagation based phased contrast imaging) instead of the more conventional radiography (near-field imaging). We use the Zyla indirect detector with 10x optical magnification, to obtain a 0.65 μm/px effective pixel size and approximately 31 μm resolution using the Fresnel number equal to unity to define discernible feature size.

The resolution limits of a microscope can be described by the component point-spread functions (PSFs). The PSFs relevant to our imaging condition (TXM) are the following:

- Detector pixelation (effective pixel size)
- Spherical aberrations of the CRLs
- Fundamental resolution limit of finite size aperture
- Chromatic aberrations of the CRLs
- Scintillator resolution during indirect detection

The detector pixelation and effective pixel size have been discussed. The smallest isolated "feature" that could be (tenuously) claimed to exist is one pixel, and the smallest line pair resolution is two pixels.

Spherical aberrations are assessed from the linearity of the TEM grids shown in Fig. 5. Due to the aberration-correcting phase plates (Seiboth *et al.*, 2017), we expect spherical and other structural aberrations to be negligible compared to other sources of blurring.

Calculations from Simons et al. (Simons *et al.*, 2017), provide our metrics for the PSF due to the physical aperture of the lens, the effective aperture due to CRL attenuation through the parabolic lenslets, and the chromatic aberration from the source bandwidth. Most of these discussions are provided in the main text.

The fundamental limit of resolution in an indirect detection scheme using scintillators has been explored in the medical and other communities (Martin & Koch, 2006). In that work, estimations are made based on the scintillator thickness and numerical aperture of the optical lenses imaging the scintillator. The Navitar Zoom 7000 lens used in this work was set to 0.8$\times$ magnification, for which it has an F-number of 16 and numerical aperture in air of approximately NA $= 1/2N$ =0.03. With a 50-μm thick scintillator as we used, an estimated 6-μm spatial resolution is expected at the

scintillator. Through the X-ray magnifying optics, this predicts roughly 150-nm spatial resolution at the object plane.

6. Temporal Resolution

The temporal resolution in this work was calibrated in three different ways to account for the different temporal information required to inform the measurement. As mentioned in previously, the temporal resolution of an image sequence must be defined based on the integration time for each frame (e.g. to inform phenomena like motion blur), and the frame rate between subsequent images that sets the dynamics that can be observed. For pump-probe experiments, another important type of temporal resolution is the precision of the delay time between the initial “pump” laser and the subsequent XFEL probes. We discuss each type of timing measurement in this section, but note that the most important measurement for timing precision in our experiment was the delay between the AM laser and the XFEL, which had the highest variability.

We first discuss the duration and possible timing jitter of the X-ray probe pulses. At the LCLS, the pulse duration may be set from 200-as to 100-fs; as our experiment did not require high-precision for the frame integration, we used the <100-fs pulse duration, which has a native timing jitter with respect to the global timing of the XFEL trigger pulse of less than ∼250-fs FWHM (Bionta *et al.*, 2014; Bostedt *et al.*, 2016). This length of time defines the “exposure time” of the imaging system and at these exposure times the atomic motion is negligible resulting in completely motion-blur free imaging (Hwu & Margaritondo, 2021). This pulse duration cannot be obtained even in one-bunch 100 ps duration synchrotron measurements, where sonic features would be in motion. Because the XFEL pulse duration and jitter are orders of magnitude less than the pulse separation or laser jitter, it is not considered further.

Our experiments were conducted in two-pulse mode (Decker *et al.*, 2022). The XFEL

pulses were separated by 2.1, 21, and 119 ns respectively, measured by fast-diode traces. The RMS jitter is ∼10 fs (Decker *et al.*, 2022). The Icarus detector could resolve these two pulses independently and with its measured 1.3 μm resolution.

The Zyla detector with resolution 0.9 μm instead integrates these two pulses together. Lower velocity features will not be discernible with these pulse separations while faster motions will "ghost" or double with sufficient contrast. These ghosting velocities depend on the pulse separation and are about 70 m/s for the 21 ns pulse separation.

As a pump-probe experiment measuring the irreversible dynamics of laser melting, the temporal stability between the pump and probe is critically important. Using the copper linear accelerator (linac) of LCLS, the probe arrival time can be controlled to increments of 350 ps (Decker *et al.*, 2022), however, the pump laser's timing stability with respect to the input TTL had significantly higher variability, as shown in Fig. 6. In Fig. 6(a), photodiode traces for many laser pulses are overlaid to characterize laser jitter relative to the X-ray pulses. Fast photodiode traces were measured with an Acqiris U1065A DC282 4-channel 2 GS/s digitizer with joined channels to increase sampling rate. This nLight AFX 1000 industrial pump laser has a trimodal dead-time shown in Fig. 6(b) between receiving the command to pulse and actually turning on of approximately 85±15 μs. The three peaks were located at 71-, 85-, and 99 μs with respectively a 16%, 63%, and 21% ratio between them. Each peak had an RMS jitter of 1.5-, 3-, and 3.5 μs jitters respectively. The rise time of all peaks in Fig. 6(c) was 5.5±0.5 μs.

The power overshoot and stabilization time was not resolved due to the noise of the photodiode measurements. The jitter in this pump "on"-time is the primary jitter in the laser melting experiment and dominates the observable features for pump-probe delays significantly less than 100 μs. However, as the laser melting process proceeds to the millisecond scale, this jitter becomes negligible and is ignored for most later

time experiments.

7. Photometrics Toolbox

The photometrics toolbox referenced in (Taylor, 2025) is a Python toolset combining the governing equations of CRL optics to predict X-ray magnification and microscope geometry, coupled to the detector parameters to estimate the resolution of a monochromatic source. It combines a wrapper of the CXRO and NIST databases to estimate sample transmissions, so that the fluence and estimated signal on the detector can also be predicted for arbitrary sample geometries and compositions, within simple TXM configurations.

The implementation of the ray-optical treatment of CRLs is derived in the work of Simons et al. (Simons *et al.*, 2017).

Fig. 7 shows the photometric calculations for our TXM microscope and detectors as a function of X-ray photon energy, given the microscope length constraints. That analysis shows the trade-offs between magnification and signal transmitted through the sample, and places bounds on the maximum X-ray photon energy allowable to obtain the desired resolution goals (considering only the pixel limited resolution).

References

Bionta, M. R., Hartmann, N., Weaver, M., French, D., Nicholson, D. J., Cryan, J. P., Glownia, J. M., Baker, K., Bostedt, C., Chollet, M., Ding, Y., Fritz, D. M., Fry, A. R., Kane, D. J., Krzywinski, J., Lemke, H. T., Messerschmidt, M., Schorb, S., Zhu, D., White, W. E. & Coffee, R. N. (2014). *Review of Scientific Instruments*, **85**(8). http://dx.doi.org/10.1063/1.4893657

Bostedt, C., Boutet, S., Fritz, D. M., Huang, Z., Lee, H. J., Lemke, H. T., Robert, A., Schlotter, W. F., Turner, J. J. & Williams, G. J. (2016). *Reviews of Modern Physics*, **88**(1). http://dx.doi.org/10.1103/RevModPhys.88.015007

Decker, F.-J., Bane, K. L., Colocho, W., Gilevich, S., Marinelli, A., Sheppard, J. C., Turner, J. L., Turner, J. J., Vetter, S. L., Halavanau, A., Pellegrini, C. & Lutman, A. A. (2022). *Scientific Reports*, **12**(1). http://dx.doi.org/10.1038/s41598-022-06754-y

Hodge, D. S., Leong, A. F. T., Pandolfi, S., Kurzer-Ogul, K., Montgomery, D. S., Aluie, H., Bolme, C., Carver, T., Cunningham, E., Curry, C. B., Dayton, M., Decker, F.-J., Galtier, E., Hart, P., Khaghani, D., Ja Lee, H., Li, K., Liu, Y., Ramos, K., Shang, J., Vetter, S., Nagler, B., Sandberg, R. L. & Gleason, A. E. (2022). *Optics Express*, **30**(21), 38405. http://dx.doi.org/10.1364/OE.472275

Hwu, Y. & Margaritondo, G. (2021). *Journal of Synchrotron Radiation*, **28**(3), 1014–1029. http://dx.doi.org/10.1107/S1600577521003325

Lehmkühler, F., Roseker, W. & Grübel, G. (2021). *Applied Sciences*, **11**(13), 6179. http://dx.doi.org/10.3390/app11136179

Martin, T. & Koch, A. (2006). *Journal of Synchrotron Radiation*, **13**(2), 180–194. http://dx.doi.org/10.1107/S0909049506000550

Seiboth, F., Schropp, A., Scholz, M., Wittwer, F., Rödel, C., Wünsche, M., Ullsperger, T., Nolte, S., Rahomäki, J., Parfeniukas, K., Giakoumidis, S., Vogt, U., Wagner, U., Rau, C., Boesenberg, U., Garrevoet, J., Falkenberg, G., Galtier, E. C., Ja Lee, H., Nagler, B. & Schroer, C. G. (2017). *Nature Communications*, **8**(1). http://dx.doi.org/10.1038/ncomms14623

Simons, H., Ahl, S. R., Poulsen, H. F. & Detlefs, C. (2017). *Journal of Synchrotron Radiation*, **24**(2), 392–401. http://dx.doi.org/10.1107/S160057751602049X

Taylor, Z., (2025). X-ray photometric toolbox. https://doi.org/10.17605/OSF.IO/KH6C5

iucr

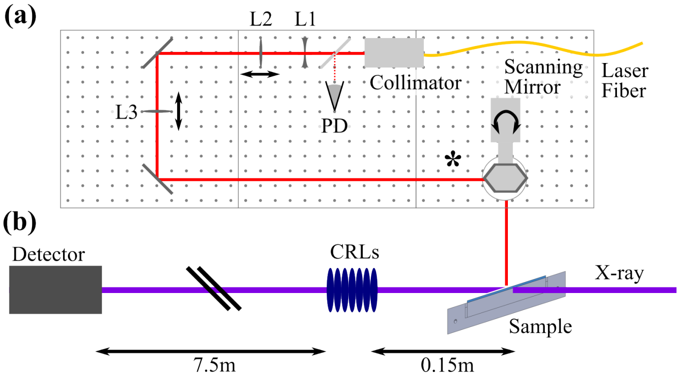

Fig. 1. (a) Laser optical path prior delivery at (b) the sample in the X-ray microscope geometry. The astric in (a) denotes the location at which the laser power was measured.

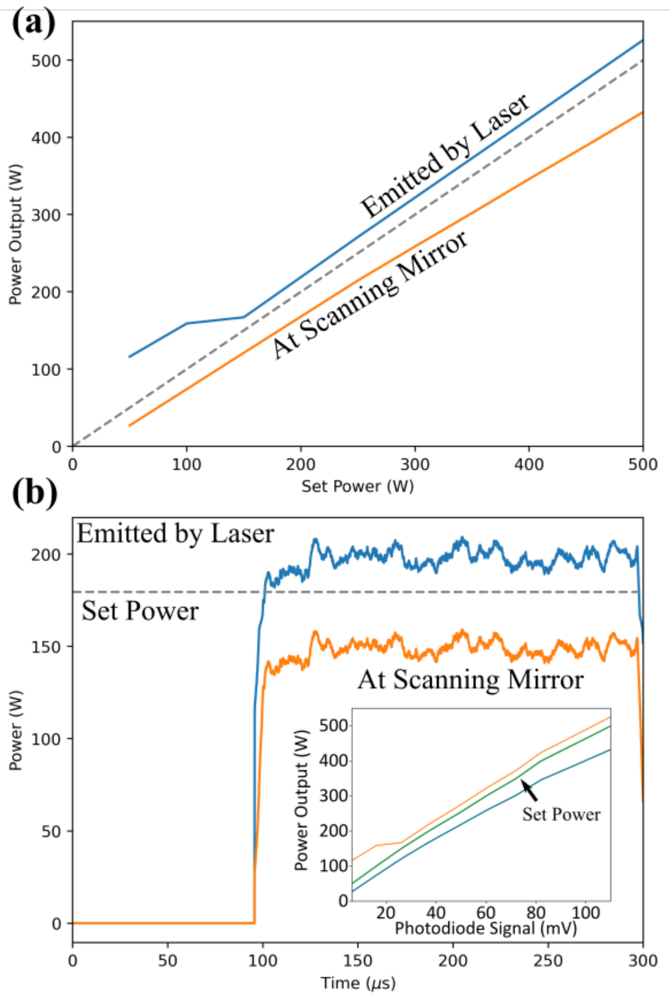

Fig. 2. (a) Power calibration between set power and both the power emitted from the fiber and reaching the scanning mirror through the optics. (b) Smoothed laser response to 180 W set power. Inset shows the photodiode to power conversion.

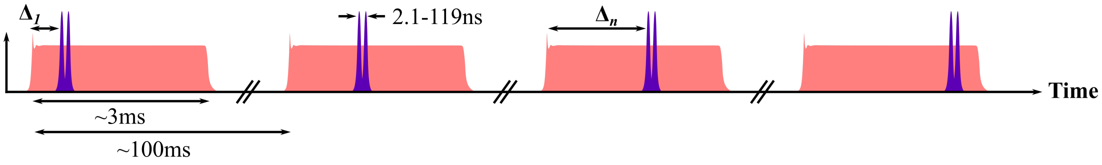

Fig. 3. Schematic of the "pump-probe" timing acquisitions used in our experiment. Red pulses denote the AM laser, while the purple pulses denote the XFEL probes (pulse durations not shown to scale).

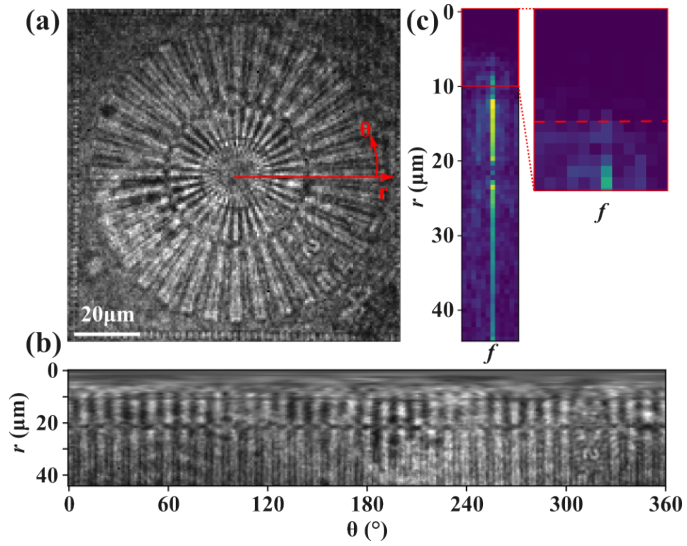

Fig. 4. (a) 90-μm Siemens resolution star imaged using the Icarus detector. (b) The polar transform of this image. (c) A subset of the Fourier transform of the polar transformed image corresponding to the Siemens star spokes with an inlay and dashed red line marking the point at which the star is no longer resolvable.

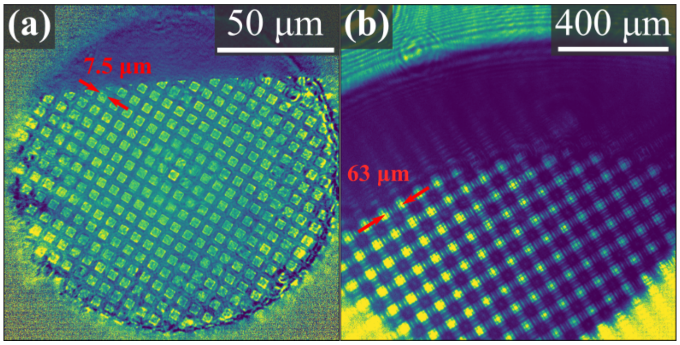

Fig. 5. TEM grid images comparing (a) TXM of a 2000-mesh grid with 31.5× magnification (39.4× magnification in X-ray and 0.8× magnification in optical) to (b) holography of a 400-mesh grid with 10× magnification only in the optical.

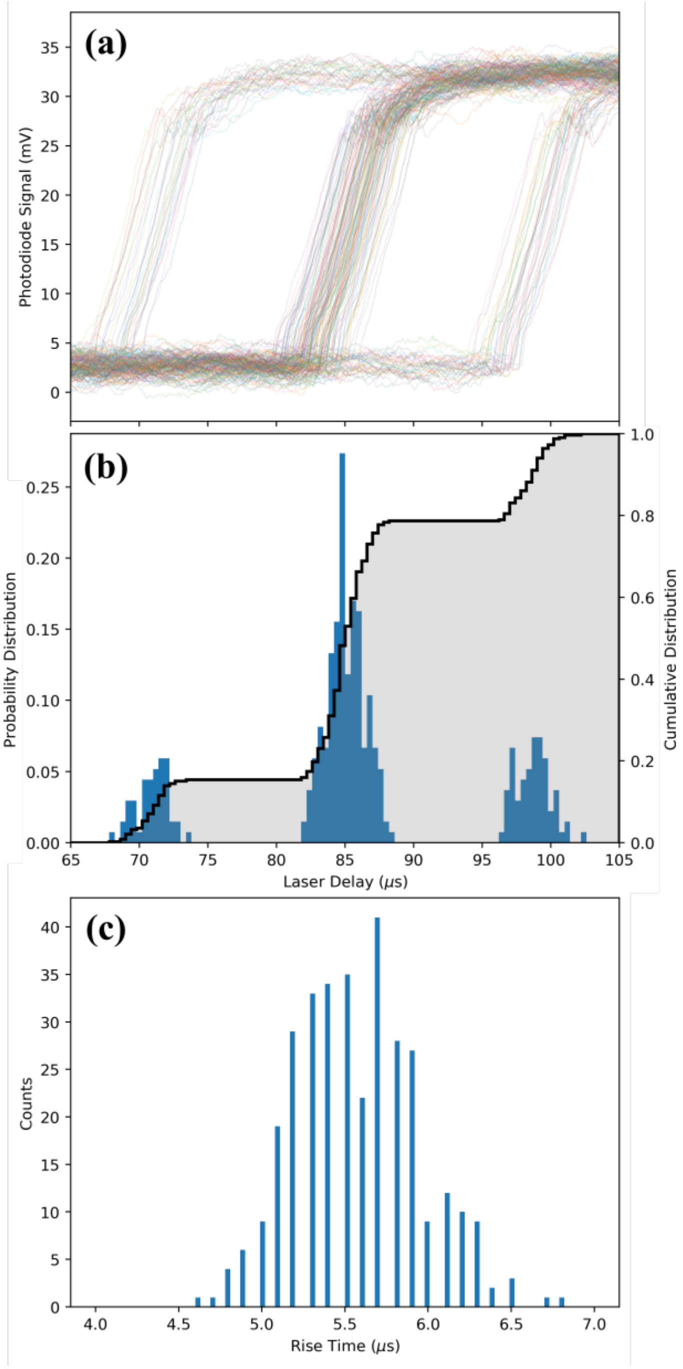

Fig. 6. (a) Overlaid, smoothed photodiode traces of laser scan over many runs and (b) population distributions of laser jitter vs time since laser trigger. (c) Population statistics of FWHM laser rise time.

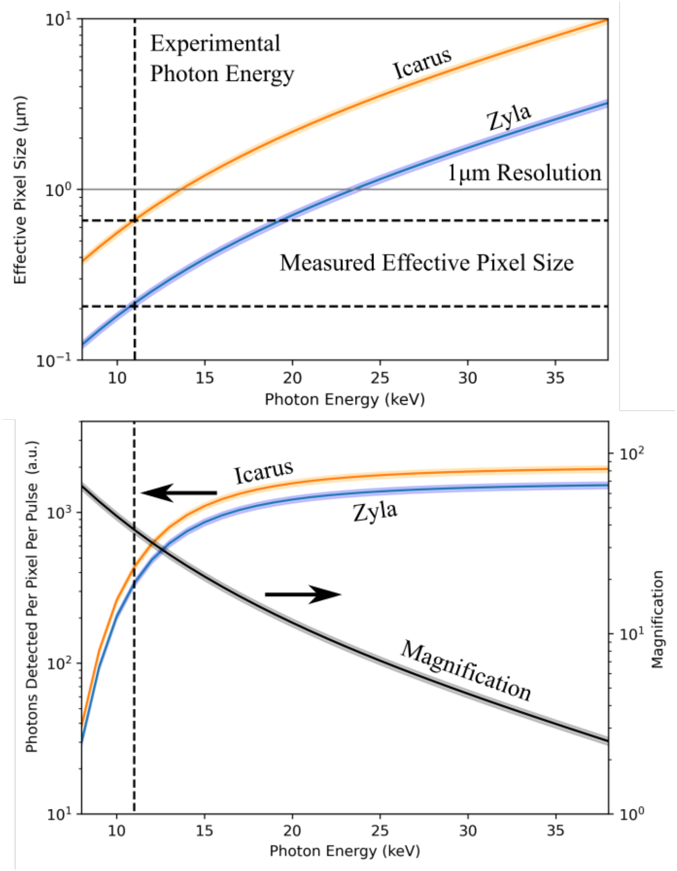

Fig. 7. Trade-offs between resolution and photon counts in the selection of X-ray energy. A 1 μm effective pixel size and detector sensitivity bounds the maximum acceptable energy. Photon counts are approximate based on SASE jitter and XFEL performance at any given moment.
